# Lipid-lowering, antihypertensive, and antithrombotic effects of nattokinase combined with red yeast rice in patients with stable coronary artery disease: a randomized, double-blinded, placebo-controlled trial

**DOI:** 10.3389/fnut.2024.1380727

**Published:** 2024-05-15

**Authors:** Man Liu, Ziyi Xu, Zongling Wang, Di Wang, Mingzhe Yang, Hui Li, Wei Zhang, Ruikun He, Huimin Cheng, Peiyu Guo, Zhongxia Li, Hui Liang

**Affiliations:** ^1^The Institute of Human Nutrition, College of Public Health, Qingdao University, Qingdao, China; ^2^BYHEALTH Institute of Nutrition & Health, BYHEALTH Co. Ltd., Guangzhou, China; ^3^Qingdao Fuwai Cardiovascular Disease Hospital, Qingdao, China; ^4^Songshan Hospital, Medical College of Qingdao University, Qingdao, China

**Keywords:** nattokinase, red yeast rice, cardiometabolic risk factors, coronary artery disease, anticoagulation, statin

## Abstract

Nattokinase (NK) and red yeast rice (RYR) are both indicated for their potential in cardiovascular disease prevention and management, but their combined effects especially in coronary artery disease (CAD) are scarcely examined. This 90-day randomized, double-blind trial aims to investigate the effect of NK and RYR supplementations on cardiometabolic parameters in patients with stable CAD. 178 CAD patients were randomized to four groups: NK + RYR, NK, RYR, and placebo. No adverse effects due to the interventions were reported. In comparisons across groups, NK + RYR showed the maximum effect in reducing triglyceride (−0.39 mmol), total cholesterol (−0.66 mmol/L), diastolic blood pressure (−7.39 mmHg), and increase in high-density lipoprotein cholesterol (0.195 mmol/L) than other groups (all p for multiple groups comparison<0.01). Both NK + RYR and NK groups had significantly better-improved lactate dehydrogenase than the others (−29.1 U/L and − 26.4 U/L). NK + RYR group also showed more potent reductions in thromboxane B2 and increases in antithrombin III compared to placebo (both *p* < 0.01). These improved markers suggest that combined NK and RYR may preferably alter antithrombin and COX-1 pathways, potentially reducing thrombosis risks in CAD patients. Overall, the combined NK and RYR supplementation is safe and more effective than separately in improving cardiometabolic markers among CAD patients with multiple heart medications use.

## 1 Introduction

Coronary artery disease (CAD) is a global epidemic disease that has been identified as the major cause of death in both industrialized and developing countries (1–3). In China, an estimated 330 million people suffer from cardiovascular diseases, including 11.39 million individuals with CAD, along with a high prevalence of hypertension and hyperlipidemia (4–6). Patients with known cardiovascular disease but have not experienced a recent acute event, such as stable angina and silent ischemia, and those who have stabilized after an acute coronary syndrome phase, are often considered as having stable CAD, who often receive long-term disease monitoring and treatment (7). Cardiometabolic conditions including hyperlipidemia, hypertension and diabetes mellitus increase the risk of CAD and predict poor CAD progression, and improving related cardiometabolic risk variables are critical strategies for CAD prevention and treatment (8–10).

Several biochemical markers are commonly used in clinical practice and research settings to evaluate patients’ cardiometabolic status and disease prognosis. Blood lipids, blood pressure, and glucose are routinely tested to monitor CAD prognosis in clinical practice. Additionally, lactate dehydrogenase (LDH) and creatine kinase (CK) are useful diagnostic markers for acute coronary syndrome (ACS), of which prolonged elevation can indicate a high risk of developing a near-future heart attack (11–13). In the prognosis of CAD, treatment is often implemented to prevent thrombosis, a complication where the formation of blood clots results in blockage of blood vessels leading to cardiovascular events (14). Antithrombin III (AT-III) plays an important role in blood clotting regulation, of which deficiency can lead to excess blood clots and subsequent thrombosis risks. Thromboxane B2 (TXB_2_) is a stable metabolite of thromboxane A2 (TXA_2_), a vasoconstrictor that stimulates platelet aggregation, while 6 − keto−prostaglandin F1 alpha (6-keto-PGF1α) is a stable metabolite of prostaglandin I2 (PGI_2_), which functions contrary to TXA_2_ and has a strong effect on capillary dilation and inhibition of platelet aggregation. TXA_2_/PGI_2_ lie on the cyclooxygenases (COX) pathway and their imbalance is one of the causes of platelet aggregation, vasospasm, or thrombosis (15).

Dietary supplements and natural extracts have been investigated for their potential or proven effect in lowering cardiometabolic risk. Nattokinase (NK), a blood-clot dissolving protein used for the therapy of cardiovascular disorders, is produced by the bacterium *Bacillus subtilis* during the fermentation of soybeans for producing natto (16). NK breaks down blood clots by directly hydrolyzing fibrin and plasmin substrate, converts endogenous prourokinase to urokinase, degrades plasminogen activator inhibitor-1, and increases tissue plasminogen activator, all of which supports its fibrinolytic activity (17). NK has been demonstrated to have fibrinolytic activity in healthy subjects, as well as in nondiabetic and hypercholesterolemic subjects (18, 19). In addition, results from *in-vitro* studies indicated that natto fractions have inhibitory effects on low-density lipoprotein-cholesterol (LDL-C) oxidation as a radical scavenger (20, 21). Still, clinical data on the effects of NK on cardiometabolic health are inconsistent. Two randomized controlled trials indicated that increased intake of NK may be effective in the management of hypertension and hyperlipidemia: increasing NK intake for 2 months resulted in favorable effects on blood pressure among patients with pre-hypertension or stage-1 hypertension (22), or blood lipids among patients with hyperlipidemia after 6-month continuous NK mono formula intervention (23). In contrast, a large-scale randomized trial with 3 years of NK intervention among elderly adults who are devoid of cardiovascular diseases indicated no significant improvements in their carotid or blood pressure measurements (24). With some evidence showing that increased intake of NK may be effective in the management of hypertension and hyperlipidemia, while a few others indicated no significant benefits in cardiometabolic indicators, it is needed to assess whether NK supplementation can be truly beneficial in the prevention or treatment of cardiometabolic diseases in various health-status (22–24).

Red yeast rice (RYR), used as a dietary supplement and herbal medicine in China for centuries, is produced by the fermentation of white rice with the yeast *Monascus purpureus*. The main functional ingredient of RYR is monacolins, in particular monacolin K, which has been confirmed to inhibit the activity of 3-hydroxy-3-methylglutaryl coenzyme A (HMG-CoA) reductase, a key enzyme in cholesterol synthesis (25). A meta-analysis including 13 randomized control trials showed that RYR significantly decreased the serum total cholesterol (TC), triglycerides (TG) and LDL-C compared with placebo (26), demonstrating the overall favorable effect of RYR on hyperlipidemia. Another systematic review and meta-analysis included 30 studies also presented findings that RYR reduces mortality and major adverse cardiovascular events, and improves biochemical parameters of blood glucose, blood lipids, and blood pressure in patients with existing cardiometabolic complications (27).

Although the use of NK and RYR separately has been widely studied in the past decades, the combined use of these two agents in CAD patients is yet insufficiently examined. In addition, cardiovascular medication use is common in this population, comprising a critical sector in conventional therapeutic strategies managing CAD, but the safety and interaction with NK or RYR intake are still rarely discussed in existing evidence (9, 16, 28). Therefore, the aim of this randomized, double-blinded, placebo-controlled trial was to investigate whether supplementation with NK and RYR can alter cardiometabolic parameters in patients with CAD and whether the combination will be superior to NK or RYR alone, as well as to examine the safety and cardiometabolic responses when co-administer with heart medications.

## 2 Materials and methods

### 2.1 Study design and participants

We ran a double-blinded, randomized, placebo-controlled study to investigate the effects of supplement combined NK with RYR on cardiometabolic parameters in patients with SCAD. This trial was done at Qingdao Fuwai cardiovascular hospital community health service center, Songshan Hospital Medical College of Qingdao University Ningxia road community health service center, Guangrao road community health service centers from January 2017 to March 2018. All patients admitted to this hospital with a diagnosis of stable CAD, were considered for participation in the study and screened by a cardiologist for eligibility, then screened to determine the research object, and signed informed consent. Inclusion criteria were: (1) confirmed CAD according to the diagnostic criteria of “Guidelines for the Diagnosis and Treatment of Chronic stable Coronary heart Disease” formulated by the Chinese Society of Cardiovascular Diseases which includes three types: chronic stable exertional angina, ischemic cardiomyopathy, and the stable course of the acute coronary syndrome with consistent medication use, or without medications; (2) no dietary supplements such as fish oil, vitamins and minerals were taken in the 3 months before screening; (3) voluntarily sign informed consent (29). Exclusion criteria were (1) acute coronary syndrome, percutaneous coronary intervention or coronary artery bypass grafting, percutaneous or surgical revascularization within the previous 3 months; (2) acute infection, or severe liver and/or kidney disease, or tumor, or peripheral vascular disease, or recently underwent a surgery; (3) patients with disabilities or showed unwillingness to comply with study treatment. All subjects gave their informed consent for inclusion before they participated in the study. The study was conducted in accordance with the Declaration of Helsinki. The study was approved by the Ethics Committee of Qingdao Center for Disease Control and Prevention (No. 201701). Trial registration: Chinese Clinical Trials Registry (ID: ChiCTR2300077936).

### 2.2 Intervention/treatment

Based on prior research, using TC, we determined that each group needs to consist of 32 participants to achieve a study power of 90% with an alpha level set at 0.05 (30). To account for an anticipated dropout rate of 20% during the trial, we plan to enroll a minimum of 40 participants in each group. Participants were randomly assigned to four interventions. Interventions were randomly coded as intervention numbers. Subjects who met the inclusion criteria were randomly assigned a unique subject identification number by research personnel and logged into the database. After the successful enrollment of the subjects, an intervention number was randomly assigned to each subject identification number. Data collection personnel, participants, and researchers performing data analysis were blinded to the intervention assignment. The assignment result is concealed as number codes selected from two sets of unrepeated numbers to be further handled by study staff and were not unveiled until the end of the trial. Participants consumed 3 placebo or test capsules after lunch and dinner daily during the whole intervention period (90 days). The capsules were provided by BYHEALTH Co., Ltd., Guangdong, China (31). The lab-tested main ingredients of the product for each group are as follows. (1) NK + RYR group: 610.3 FU NK and 1.5 mg monacolins per capsule; (2) NK group: 602.5 FU nattokinase per capsule; (3) RYR group: 1.5 mg monacolins per capsule; (4) Placebo group: maltodextrin powder. Accordingly, the treatment for NK + RYR, NK, and RYR group was 3661.8 FU NK and 9.0 mg monacolins, 3615.0 FU NK alone, and 9.0 mg monacolins alone per day, respectively. The capsules had identical packaging with no differences in appearance, texture, and smell, so participants and researchers were masked from the groups assigned and capsules given. Participants were instructed to resume their normal diet and medication regimens throughout the trials.

### 2.3 Data collection and assessment

Patients’ demographic information, lifestyle factors including smoking and alcohol consumption, and medical history including medication use were obtained during a face-to-face interview with a trained researcher. Dietary intake was assessed using a 3-day, 24-h dietary recall, and nutrient values were calculated using the integrated Traditional Chinese and Western Medicine Nutritional Therapy Computer Expert System MX2 version (NCCWMX2/NCCW12.0). To assess anthropometrics, all participants had been requested to take off outerwear and shoes when measuring body height and body weight. Body height and weight were measured by ultrasonic height and weight meter (Woshen, WS-H200). Blood pressure and heart rate were measured at baseline and after intervention by electronic sphygmomanometer (OMRON, HBP9020) when the participants kept calm in a quiet environment.

Blood samples were collected from all participants at baseline and endpoint. 8 milliliters of blood had been drawn from the antecubital fossa. Samples were centrifuged at 4°C at 3000 rpm for 15 min and the serum had been removed and stored in −80°C for subsequent analysis. Serum lipids including TG, TC, LDL-C, high-density lipoprotein cholesterol (HDL-C), and blood glucose were evaluated using the enzymatic method by automatic biochemical analyzer test. LDH and CK were determined by enzyme-linked immunosorbent assay (ELISA). 108 participants’ blood samples were tested for ELISA markers TXB2, 6-keto-PGF1α, and AT-III. All ELISA test kits were purchased from Jianglai Biological Technology Co., Ltd. (Human Thromboxane B2 (TXB2) ELISA kit JL12478; Human 6-keto-Prostaglandin F1 Alpha (6-keto-PGF1a) ELISA Kit JL46688; Human Antithrombin III (AT-III) ELISA Kit JL19564). All blood preparation measurements were carried out according to the manufacturer’s instructions.

### 2.4 Statistical analysis

Continuous variables are described as mean (standard deviation) while categorical variables are presented as percentages. The chi-square test was used to compare the categorical variables among groups. Shapiro–Wilk test was performed for all continuous variables to assess normality. Paired *t*-test was used to compare parameters between baseline and day 90 in each group. For continuous variables that are normally distributed, ANOVA was used to compare parameters among groups, and Fisher’s Least Significant Difference (LSD) test was used between each group. For non-normally distributed variables, Kruskal-Wallis tests were performed among all groups and Dunn’s tests were used between each group. A two-tailed *p*-value of <0.05 was considered statistically significant. All statistical analyses were carried out with R version 4.3.1 (32).

## 3 Results

### 3.1 Baseline characteristics of the participants

**Figure 1:**
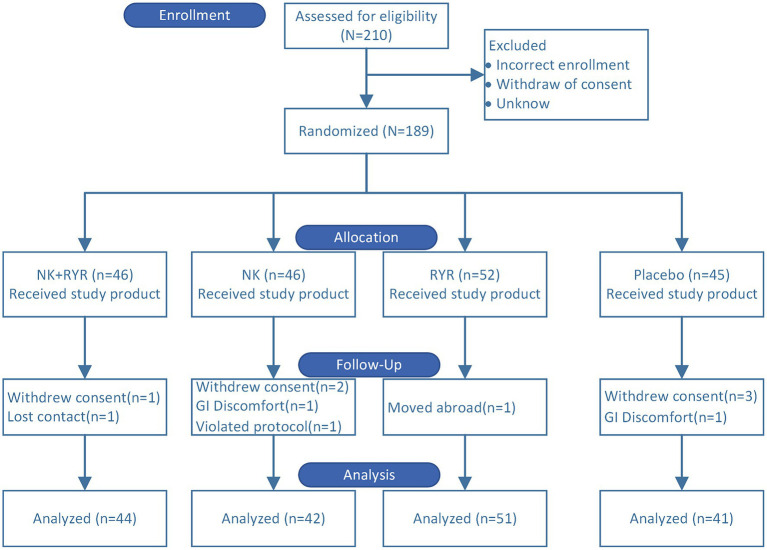
Consolidated standards of reporting trials participant flow for the randomized trial. A total of 210 patients were screened and 189 eligible patients were randomized to four intervention groups. 11 participants dropped out during the trial. 178 participants were included in the final analysis.

In total, 189 stable CAD patients who fulfilled the inclusion and exclusion criteria were enrolled in the study and randomized to one of the four treatment groups. During the trial period, 11 participants were lost during follow-up (5.8%), including 6 who refused to return to visit, 2 who were unable to contact due to moving or traveling abroad, 1 who took other health care drugs without permission, 2 who stopped taking capsules due to experiencing adverse events that resulted in discontinuation. In specific, the two incidences of adverse events were abdominal discomfort: one in the placebo group and one in the NK group, respectively, which were probably not related to the study intervention. A total of 178 participants were finally included in the analysis: NK-RYR group (*n* = 44), NK group (*n* = 42), RYR group (*n* = 51) and placebo group (*n* = 41) (Figure 1).

The general characteristics of the four groups at baseline are summarized in Table 1. The four groups were balanced in baseline parameters including age, gender, body mass index (BMI), CAD family history, drinking and smoking, and related medication use (statin, *β*-blockers, aspirin, and Ace inhibitors). For biochemical measurements, the RYR group had a higher baseline fasting glucose than the rest, and the placebo control group had a marginally higher HDL-C compared to the intervention groups. The nutrient values from the mean of the 3-day, 24-h recall, as well as the exercise minutes, were balanced among groups and remained consistent throughout the trial (Supplementary Table S2). Among 108 participants whose blood samples were tested for ELISA anticoagulation markers, all baseline characteristics were balanced across the four treatment groups except for blood glucose (Supplementary Table S3).

**Table 1:** Baseline characteristics of the study participants.

	NK-RYR group	NK group	RYR group	Placebo	*p* value*
*n*	44	42	51	41	
General					
Men (%)	15 (34.1%)	11 (26.2%)	25 (49.0%)	20 (48.8%)	0.0710
Age (years)	64.3 (8.42)	61.0 (9.87)	60.7 (8.67)	63.0 (8.13)	0.2022†
BMI (kg/m^2^)	26.2 (2.72)	26.0 (3.08)	26.3 (2.85)	25.6 (2.55)	0.4781†
CAD family history (%)	14 (31.8%)	17 (40.5%)	15 (29.4%)	19 (46.3%)	0.3181
Drinkers (%)	11 (25.0%)	9 (21.4%)	7 (13.7%)	7 (17.1%)	0.5318
Smokers (%)	14 (31.8%)	8 (19.0%)	6 (11.8%)	9 (22.0%)	0.1174
Biochemical					
SBP (mm Hg)	140 (19.2)	142 (12.0)	138 (15.4)	140 (17.7)	0.6851
DBP (mm Hg)	82.3 (12.1)	82.4 (6.71)	79.4 (8.62)	80.3 (12.7)	0.1524†
TG (mmol/L)	1.77 (0.483)	1.71 (0.396)	1.69 (0.641)	1.70 (0.461)	0.5235†
TC (mmol/L)	5.50 (0.891)	5.07 (0.933)	5.19 (0.995)	5.50 (0.684)	0.0549
HDL-C (mmol/L)	1.36 (0.190)	1.29 (0.323)	1.33 (0.292)	1.43 (0.268)	0.0437†
LDL-C (mmol/L)	3.20 (0.952)	3.15 (0.911)	3.19 (0.789)	3.28 (0.565)	0.8958
Glucose	6.10 (1.48)	6.10 (1.23)	6.89 (1.66)	5.84 (2.16)	< 0.0001†
Medications					
Statin (%)	28 (63.6%)	23 (54.8%)	31 (60.8%)	26 (63.4%)	0.8239
Aspirin (%)	11 (25.0%)	8 (19.0%)	11 (21.6%)	12 (29.3%)	0.7107
β Blocker (%)	11 (25.0%)	11 (26.2%)	17 (33.3%)	17 (41.5%)	0.3369
ACE Inhibitor (%)	11 (25.0%)	10 (23.8%)	9 (17.6%)	8 (19.5%)	0.8014

Continuous variables are presented as mean (SD), categorical variables are presented as the number of people who answered yes (%).

NK-RYR, a supplement of nattokinase and red yeast rice; NK, nattokinase; RYR, red yeast rice; BMI, body mass index; CAD, coronary artery disease; SBP, systolic blood pressure; DBP, diastolic blood pressure; TG, triglycerides; TC, Total cholesterol; HDL-C, high-density lipoprotein cholesterol; LDL-C, low-density lipoprotein cholesterol. *ANOVA for continuous variables with normal distribution, Kruskal-Wallis test for continuous variables with non-normal distribution (*p* value marked †), Chi-square test for categorical variables.

### 3.2 Blood lipids

TG, TC, HDL, LDL, and glucose were indicators included in the blood lipids evaluation of our study (Table 2). In within-group comparisons, the NK + RYR group had significant improvements in all indicators after 90 days of intervention (TG −0.392 mmol/L, *p* < 0.0001; TC -0.661 mmol/L, *p* < 0.0001; LDL-C -0.537 mmol/L, *p* < 0.0001; HDL-C + 0.195 mmol/L, *p* < 0.0001), while the placebo group only had small improvements in TC (−0.0449 mmol/L, *p* = 0.0041) and HDL-C (+0.0615 mmol/L, *p* = 0.0173). NK group had significant improvement in all indicators except HDL-C, and RYR group improved in all except TG.

**Table 2:** Plasma lipid, glucose concentrations, and blood pressure at baseline and after 90 days of intervention.

Lipid parameter	Treatment	Baseline (T0)	90 Days (T1)	*p* value**	Change (T1-T0)	
					mmol/L or mmHg	%
TG (mmol/L)	NK-RYR	1.77 (0.483)	1.38 (0.466)	< 0.0001†	−0.392 (0.459)^a,c^	−19.5 (27.2)^a,b,c^
	NK	1.71 (0.396)	1.47 (0.412)	< 0.0001†	−0.236 (0.316)	−12.6 (17.3)
	RYR	1.69 (0.641)	1.56 (0.551)	0.1538†	−0.121 (0.599)	−1.40 (30.8)
	Placebo	1.70 (0.461)	1.59 (0.604)	0.1197†	−0.112 (0.451)	−5.79 (27.2)
	*p* value*	0.5235†	0.1453†		0.0028	0.0033
TC (mmol/L)	NK-RYR	5.50 (0.891)	4.84 (0.794) ^a^	< 0.0001	−0.661 (0.642)^a,b^	−11.4 (11.2)^a,b^
	NK	5.07 (0.933)	4.75 (0.887) ^a^	0.0038	−0.321 (0.679)	−5.73 (12.0)^a^
	RYR	5.19 (0.995)	4.78 (0.977) ^a^	0.0002	−0.407 (0.729)^a^	−7.08 (12.5)^a^
	Placebo	5.50 (0.684)	5.45 (0.649)	0.0041	−0.0449 (0.0944)	−0.725 (1.84)
	*p* value*	0.0549	0.0003		< 0.0001	<0.0001
LDL-C (mmol/L)	NK-RYR	3.20 (0.952)	2.66 (0.791) ^a^	< 0.0001	−0.537 (0.600)^a^	−14.6 (18.2)^a^
	NK	3.15 (0.911)	2.80 (0.909) ^a^	< 0.0001	−0.352 (0.504)^a^	−10.9 (16.1)^a^
	RYR	3.19 (0.789)	2.66 (0.806) ^a^	< 0.0001	−0.538 (0.626)^a^	−16.0 (17.0)^a^
	Placebo	3.28 (0.565)	3.27 (0.537)	0.4937	−0.00976 (0.0904)	−0.129 (2.81)
	*p* value*	0.8958	0.0006		< 0.0001	< 0.0001
HDL-C (mmol/L)	NK-RYR	1.36 (0.190)	1.55 (0.194) ^b,c^	< 0.0001†	0.195 (0.227)^a,b,c^	16.1 (19.6)^a,b,c^
	NK	1.29 (0.323)	1.30 (0.317) ^a,c^	0.8811†	0.00738 (0.318)	3.48 (24.9)
	RYR	1.33 (0.292)	1.43 (0.265) ^b^	0.0062†	0.103 (0.257)	10.1 (21.0)
	Placebo	1.43 (0.268)	1.49 (0.194)	0.0173†	0.0615 (0.159)	5.71 (11.8)
	*p* value*	0.0437†	< 0.0001†		0.0053	0.0067
Glucose (mmol/L)	NK-RYR	6.10 (1.48)	5.80 (1.82)	0.0128†	−0.303 (0.774) ^a,b,c^	−5.57 (11.9) ^b,c^
	NK	6.10 (1.23)	5.31 (1.13)	< 0.0001†	−0.791 (1.05) ^a^	−12.0 (13.7) ^a^
	RYR	6.89 (1.66) ^a^	5.78 (1.84)	< 0.0001†	−1.11 (1.41) ^a^	−16.0 (18.2) ^a^
	Placebo	5.84 (2.16)	5.85 (2.10)	0.9351†	0.00220 (0.171)	0.279 (2.05)
	*p* value*	<0.0001†	0.4923†		< 0.0001	< 0.0001
SBP (mmHg)	NK-RYR	140 (19.2)	130 (17.7)	< 0.0001	−9.50 (7.25)^a^	−6.64 (5.03)^a^
	NK	142 (12.0)	135 (11.0)	< 0.0001	−6.33 (7.30)	−4.32 (5.05)
	RYR	138 (15.4)	131 (12.8)	< 0.0001	−6.94 (8.84)	−4.68 (6.51)
	Placebo	140 (17.7)	137 (18.8)	< 0.0001	−3.78 (4.34)	−2.78 (3.18)
	*p* value*	0.6851	0.1235		0.0047	0.0001
DBP (mmHg)	NK-RYR	82.3 (12.1)	74.9 (11.7) ^b,c^	< 0.0001†	−7.39 (7.60)^a,b,c^	−8.69 (8.22)^a,b,c^
	NK	82.4 (6.71)	83.4 (7.39)	0.3980†	0.98 (7.41)	1.49 (8.92)
	RYR	79.4 (8.62)	80.9 (11.0)	0.1861†	1.53 (8.15)	2.05 (10.5)
	Placebo	80.3 (12.7)	80.1 (10.8)	0.8286†	−0.20 (5.73)	0.48 (8.78)
	*p* value*	0.1524†	0.0019†		< 0.0001	< 0.0001

Values are shown as means (SD).

* *p* value among groups analyzed by ANOVA if normally distributed, or Kruskal-Wallis if non-normal (*p* value marked †); ** *p* value between baseline and 90 days of intervention analyzed by paired t-test if normally distributed, or Wilcoxon test if non-normal (*p* value marked †), both adjusted using Benjamini-Hochberg method. ^a^ *p* < 0.05 compared with placebo, ^b^ *p* < 0.05 compared with NK group, ^c^ *p* < 0.05 compared with RYR group, analyzed by LSD test (normal distribution) or Dunn’s test (non-normal distribution).

For across-group comparisons, the change in lipid levels showed statistically significant differences among 4 groups (all *p* < 0.01). *Ad-hoc* analysis comparing two-group combinations reveals more detailed differences: compared with the placebo group, the NK + RYR group had significantly larger reductions in all lipid markers (all *p* < 0.01), while the NK group only showed an advantage over the placebo group in LDL-C (*p* = 0.01), and the RYR group showed an advantage over the placebo group in TC (*p* = 0.02) and LDL-C (*p* < 0.01). Additionally, the NK + RYR group showed significantly higher magnitudes of improvements in TG (*p* < 0.01) and HDL-C (*p* = 0.03) than the RYR group; and significantly higher magnitudes of improvements in TC (*p* = 0.04) and HDL-C (p < 0.01) than the NK group (Significant difference marked as a,b,c super-scripts in Table 2, exact *p* not shown in the table).

### 3.3 Blood pressure and glucose

We’ve collected baseline and 90-day SBP and DBP to reflect blood pressure status and changes in our participants (Table 2). Promisingly, the blood pressure means of each group after the 90-day intervention has all fallen to high-normal compared to grade 1 hypertension at baseline. For within-group comparisons, all groups showed significant improvements in SBP after the 90-day trial period (NK + RYR: −9.5 mmHg, NK: −6.33 mmHg, RYR: −6.94 mmHg, Placebo: −3.78 mmHg; all *p* < 0.001), but only NK + RYR group showed improvement in DBP level (−7.39 mmHg, *p* < 0.001). The magnitude of blood pressure lowering across 4 groups showed statistical significance (*p* < 0.01 for both SBP and DBP). The NK + RYR group resulted in a significantly larger SBP reduction than the placebo group (*p* < 0.01), and significantly larger DBP reduction than all other groups (all *p* < 0.01).

Comparing the glucose level changes, all supplemental groups showed significantly larger reductions than the placebo group (NK + RYR: −0.303 mmol/L, *p* = 0.02 compared to placebo; NK:-0.791 mmol/L, *p* < 0.01 compared to placebo; RYR:-1.11 mmol/L, *p* < 0.01 compared to placebo). Both the NK group and RYR group had a higher magnitude of reduction than the NK + RYR group.

### 3.4 ACS and safety parameters

LDH and CK were included as cardiac biomarkers for ACS, with additional UA assessed to monitor safety (Table 3). Upon reviewing within-group changes, we have observed significant reductions of both LDH and CK in all groups after 90 days of the intervention period (for LDH all *p* < 0.01, for CK all *p* < 0.0001). When comparing the magnitude of change across groups, NK + RYR group and NK group had greater reductions (NK + RYR:-29.1 U/L, NK:-26.4 U/L) than the other two groups (RYR:-12.8 U/L, Placebo: -10.9 U/L) only in LDH, while no across-group difference was found in the CK change magnitude.

**Table 3:** Lactate Dehydrogenase, Creatine kinase, and uric acid at baseline and after 90-day intervention.

Enzyme parameter	Treatment	Baseline (T0)	90 Days (T1)	*p* value**	Change (T0-T1)	
					U/L	%
LDH (U/L)	NK-RYR	181 (27.3)	152 (41.2) ^a,c^	< 0.0001	−29.1 (33.7) ^a,c^	−16.0 (19.3)^a,c^
	NK	185 (21.0)	159 (23.1)	< 0.0001	−26.4 (16.6) ^a,c^	−14.2 (9.18)^a,c^
	RYR	182 (23.6)	169 (33.8)	0.0026	−12.8 (29.0) ^b^	−6.84 (15.3) ^b^
	Placebo	186 (28.8)	175 (34.7)	0.0022	−10.9 (21.3)	−5.80 (12.0)
	*p* value*	0.7480	0.0083		0.0006	0.0009
CK (U/L)	NK-RYR	103 (24.9)	94.2 (26.0) ^a^	< 0.0001	−9.09 (12.8)	−8.44 (11.9)
	NK	112 (31.0)	98.4 (29.1)	< 0.0001	−13.7 (17.3)	−11.0 (14.5)
	RYR	111 (32.2)	97.7 (27.9)	< 0.0001	−13.8 (18.5)	−10.8 (16.2)
	Placebo	120 (22.8)	109 (24.1)	< 0.0001	−11.2 (14.1)	−9.04 (11.3)
	*p* value*	0.0608	0.0433		0.4427	0.7629
UA (U/L)	NK-RYR	317 (77.5)	308 (74.7)	0.2789	−9.25 (56.0) ^c^	−1.83 (14.5) ^c^
	NK	312 (72.6)	305 (69.3)	0.0192	−6.95 (18.5) ^a,c^	−1.92 (6.09) ^a,c^
	RYR	333 (58.7)	309 (56.1)	< 0.0001	−24.4 (30.4) ^a,b^	−6.92 (9.09) ^a,b^
	Placebo	313 (78.4)	314 (76.6)	0.2270	1.54 (8.02)	0.705 (2.70)
	*p* value*	0.1545	0.8762		< 0.0001	< 0.0001

Values are shown as means (SD).

LDH, lactate dehydrogenase; CK, creatine kinase; UA, uric acid. * *p* value among groups analyzed by ANOVA; ** *p* value between baseline and 90 days of intervention analyzed by paired *t*-test, adjusted using Benjamini-Hochberg method. ^a^ *p* < 0.05 compared with placebo, ^b^ *p* < 0.05 compared with NK group, ^c^ *p* < 0.05 compared with RYR group, analyzed by LSD test (normal distribution) or Dunn’s test (non-normal distribution).

UA was included for evaluation of safety, as well as a factor involved in salt-sensitive hypertension. Only the NK group and RYR group had decreased UA levels after the 90-day intervention compared to their respective baseline (NK:-6.95, *p* = 0.019; RYR:-24.4, *p* < 0.0001). Change magnitude also showed significant differences across 4 groups, but the individual variation is high in each group as suggested by the high standard deviations (Table 3).

### 3.5 Anticoagulation properties: plasma TXB2, 6-keto-PGF1α, and AT-III levels

**Figure 2:**
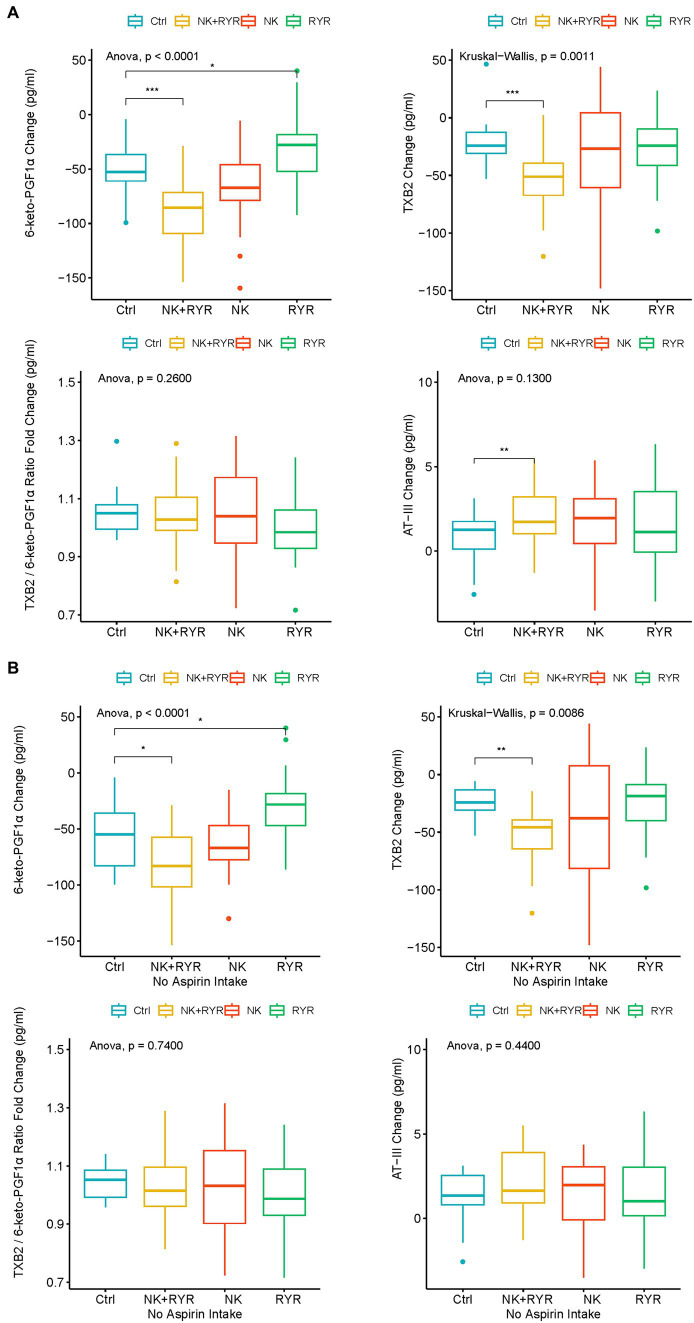
Change in 6-keto-prostaglandin F1 alpha (6-keto-PGF1α), thromboxane B2 (TXB2), TXB2/6-keto-PGF1α ratio, antithrombin III (AT-III) among intervention groups. **(A)** Comparison among all participants in intervention groups; **(B)** Comparison among participants who had no aspirin intake in the intervention groups. The box plots show averages and interquartile ranges of the measured biomarkers’ changes. *p < 0.05, **p < 0.01, and ***p < 0.001.

In our analysis of blood coagulation markers, we have found that both TXB2 and 6-keto-PGF1α levels have decreased, while AT-III levels have increased in all groups after the 90-day intervention compared to baseline levels (Figure 2A). In comparisons of the change magnitudes across groups, we found that compared to the placebo group, the NK + RR group showed a significantly larger decrease in both TXB2 and 6-keto-PGF1α levels (*p* < 0.001 for both), and a significantly larger increase in AT-III (*p* = 0.0038). Compared to the placebo group, the RR group had a smaller decrease in the 6-keto-PGF1α level (*p* = 0.025). To assess the balance between TXA2 and PGI2, we calculated the ratio of TXB2/6-keto-PGF1α, and observed neither within-group post-intervention change nor among-group difference in the balance (Figure 2A).

Considering that Aspirin has a profound inhibitory effect directly on prostaglandin and TXA2 synthesis, we further did a sensitivity analysis on TXB2 and 6-keto-PGF1α in only participants without aspirin use during the trial (*n* = 136, ELISA samples available from 79 participants). We found that the larger reduction in TXB2 and 6-keto-PGF1α remained significant in the NK + RYR group compared to the placebo (*p* = 0.009 for TXB2, *p* = 0.013 for 6-keto-PGF1α), and 6-keto-PGF1α level in RYR group marginally differ from the placebo group (*p* = 0.04). NK group did not differentiate significantly from the placebo group in the change of these two markers. The balance of TXA2 and PGI2 indicated by the TXB2/6-keto-PGF1α ratio still remains insignificant in both post-intervention changes and among group differences in the no aspirin sub-group (Figure 2B).

### 3.6 Blood lipids, blood pressure, and glucose in subgroups with statin use

In consideration of statin’s profound effect on blood pressure and blood lipids especially LDL-C, we conducted a subgroup analysis separating those who used statin (*n* = 108) during intervention from those who did not (*n* = 70) (Supplementary Table S5). We’ve found a greater magnitude of all blood lipids, blood pressure, and glucose markers improvements in participants who use statin, than those without statin use; and the across-group change advantage observed in supplement groups compared to placebo presented above remains significant. In participants without statin use, only DBP, TC, and LDL in the NK + RYR group remained significantly larger improvements when compared to the placebo group. Still, the trends of improvements were observed for other markers, and the absent significance may be due to insufficient sample size to detect the significance.

## 4 Discussion

In the present randomized, parallel control trial among patients with stable CAD, we signified differences in the magnitude of improvement between combined and single supplementations of NK and RYR, highlighting the benefits of using combined supplementation in magnifying the favorable biomarkers change in CAD disease management. This primary question of investigating the difference between single and combined supplementation in the CAD population sets our study apart from previous trials using a placebo as the only comparison group. Specifically, the NK + RYR group showed more profound improvements compared to the placebo in all blood pressure and lipid indicators, while the other two single supplementation groups only had a few biomarkers improved more than the placebo, suggesting that the combination of these two substances could potentially exert a magnified blood pressure and lipid-lowering effects in the CAD population.

We observed that all groups were having lipid-lowering results post-intervention, only that their magnitudes were different, which is partially consistent with prior research in that combined NK and RYR exert more potent effects on blood lipids than NK alone, but the NK group was not observed to be effective in previous research (23). This could partly be due to the participants’ disease difference between the two studies, where our participants were bearing stable CAD instead of dyslipidemia in the previous study, which may respond differently to NK supplementations. We also found HDL-C and TG benefits in the combined supplementation, which was not significant in a recent study using the same supplementation for 4 months (33); likewise, study participants were CAD patients in our study whereas the previous trial by Liu et al. recruited patients diagnosed with dyslipidemia but not CAD, therefore may have different physiological effects after the intervention. In studies with NK alone, another small pilot study by Wu et al. used a higher nattokinase dosage (4,000 Fu) but in a relatively short trial period (8 weeks) did not observe significant changes in lipid markers (34), and Chen et al. found improved lipid profile only with 10,800 FU/d regimen but not 3,600 FU/d in a 12-month trial among patients who were hyperlipidemic (35). These findings together suggest that using a robust dose supplementation regimen could potentially introduce significant effects on more markers over a prolonged period, while when NK and RYR are combined, the effective dosage is lower and requires a shorter time length than using them separately. Additionally, the effective dosages and length of intake may slightly differ between patients with CAD versus hyperlipidemia alone. While as of current, the upper dosage and length for safe and efficient intake of NK, RYR or combined are not yet determined, our regimen produced no reported adverse effects in CAD patients, which may encourage future exploration of higher dosages to trigger more robust physiological benefits. Regarding glucose metabolism, our results suggest that combined NK and RYR may potentially reduce blood glucose levels. Such benefits were not observed in previous research where most participants’ blood glucose was in the normal range (33). Still, validations of such effects are needed in future studies. In consideration that the baseline glucose level was slightly imbalanced among groups, we adjusted the baseline glucose to analyze the primary outcomes, and the observed improvements in blood lipids and blood pressure remained significant (data not shown).

Regarding our study population targeting patients with stable CAD, a factor to consider is their risk of ACS, of which LDH and CK were diagnostic markers (12). Promisingly, all groups had reduced LDH and CK after the 90-day trial period. While comparing the level of reduction among groups, the NK + RYR group and NK group are better at reducing LDH than the other groups, and the NK + RYR group had a larger reduction than the NK group. These findings suggest that there might be boosted effects of reducing ACS risks using combined supplementation in regular CAD disease management. To our knowledge, prior studies have not published LDH and CK results with NK and RYR supplementations. The indication of favorable LDH and CK changes in CAD patients first seen in the present study reveals the exciting potential of the effects of NK and RYR in preventing acute cardiovascular events, aside from most studied longer-term lipid and blood pressure-related chronic clinical manifestations.

Blood coagulation properties are critical in monitoring CAD disease progression and thrombosis prevention (36). Direct blood coagulation is regulated by AT-III, a physiological anticoagulant, to avoid abnormal clotting and subsequent thrombosis risk, while vascular control involves the balance between vasodilation through PGI_2_ (COX-2 pathway) and vasoconstriction through TXA_2_ (COX-1 pathway) (15). We measured AT-III, 6-keto-PGF1α and TXB_2_ which are PGI_2_ and TXA_2_ metabolites respectively, to reflect any changes in the blood coagulation and vascular response with interventions and the general management during the 90-day trial period. We observed a favorable direction of change among all participants in AT-III and TXB_2_, while an opposite direction of change in 6-keto-PGF1α. Such directions of change are still observed after excluding participants taking aspirin in the sensitivity analysis. NK + RYR group showed a significant advantage in AT-III anticoagulation activities, as well as down-regulating COX-1 related vasoconstriction over other groups regardless of aspirin use. While we are unable to compare our results on TXB_2_ and 6-keto-PGF1α since there was no prior reporting, our result on AT-III is consistent with previous data showing a single-dose NK administration increased blood antithrombin concentration 2 and 4 h post-administration (37). These results indicate that the potential triggering of anti-coagulation was only confirmed in the inhibition of vasoconstriction through the COX-1 pathway and AT-III’s direct anticoagulation, while the reason why all participants had inversed COX-2 regulation was yet unclear. Extending research is needed to understand factors contributing to the process and explain the changes in the COX-2 pathway. Collectively, results of early and sensitive implications from anticoagulation factors may indicate possible mechanisms to produce antithrombotic effects with NK and RYR supplementations.

Although we did not exclude participants with common heart medication use, the percentages of participants taking each inquired medication were balanced across the four groups, and participants had consistent drug regimens during the trial, which makes the study outcomes comparable. Notably, the overall favorable lipids and blood pressure improvements in all groups could be partially attributed to the medical management of CAD. Considering that heart medications are commonly prescribed in the management of CAD, we think it would be valuable to assess the combined effect of NK and RYR supplementation with these medications as well as the safety and tolerances, to shed light on the indication of using these supplements in the diseased population undergoing conventional care. This research question is novel, since prior studies with similar dosages in the combined NK and RYR were assessed in people without statin use or not reported medication use (23, 33). There was one study that added red yeast rice to statin treatment and concluded the general safety (28). In our present study, no adverse events were reported due to the concurrent use of heart medications with our interventions, suggesting that using supplementation with our tested dosages while continuing usual care would be a considerable option to optimize the clinical outcomes. In assessing the combined effectiveness of interventions and medications, while subgrouping led to a smaller sample size and weaker ability to detect significant differences, we still observed an advantage in the NK + RYR group in improving all blood pressure and lipid markers in participants with statin use compared to other groups. Although in participants without statin use, we have lost such significant advantage in SBP, TG, HDL-C, and glucose, it is likely due to inadequate sample size, and we still see the trends of better improvement than the placebo. We envision that in future design more people with statin use can be enrolled to assess any interactions between these agents in producing favorable clinical outcomes.

Several aspects of the design are strengths of our study. We assessed the combined use of NK and RYR with positive comparisons of the single supplementations as well as placebo control. This design enabled us to assess if there may be added benefits from combining these two substances on cardiometabolic markers. We conducted the study in stable CAD participants, whose needs to improve cardiometabolic conditions are exceedingly higher than healthy or marginally high-risk people to prevent recurrence of stroke. Our inclusion of participants concurrently taking common cardiovascular-related medications improved the generalizability of the study, as excluding conventional pharmacotherapies in real-life CAD management is often unrealistic. Moreover, our design examining if it’s safe and effective to concurrently use NK and RYR supplementations with medications provided evidence for usage recommendations in the CAD diseased populations. Besides the most assessed lipid and blood pressure indicators, we included sensitive plasma biomarkers to explore NK and RYR’s potential involvement in blood clotting and thrombosis risks, as well as markers for ACS further complementing the full scope of how interventions and management can contribute to the closed loop of long-term disease management as well as acute events prevention during the course of CAD. Additionally, the length of our intervention was 3 months, which was relatively long compared to most of the previous trials, reflecting the long-term physiological effects produced by NK and RYR interventions.

One limitation of our study is the sample size once subgrouping our participants by medication use, resulting in a reduced ability to detect significant differences due to inadequate participants in each group. Also, the observed physiological improvement may be affected by medications, but the medication uses were consistent among our participants, and the groups were balanced, which helped validate the comparisons. In future studies, it would also be beneficial to include more comprehensive data collection on previous disease courses to enhance our understanding of its relationship with supplement effects and cardiometabolic responses. Due to resource limitations, we did not perform ELISA analysis of thrombosis-related markers in all participants, but the profile of the participants with complete ELISA data was not different than the entire sample. Although we had multiple groups by supplement types and combinations, we did not examine varied dosages in each group. Also, the lack of midterm blood measurement limited our understanding of the overall timing of effective results and more subtle changes in the coagulation markers change. We are hoping that future studies with more available resources could consider these limitations and complement with additional evidence.

## 5 Conclusion

In conclusion, the present study demonstrated that combined supplementation of NK and RYR for 3 months was more effective in improving cardiometabolic parameters than using NK or RYR alone, or using standard management strategies alone, in patients with stable CAD. The combined supplementation showed an advantage in anti-coagulation activities from increased antithrombin concentration, and down-regulating COX-1 related vasoconstriction apart from aspirin use, suggesting its promising potential for thrombosis prevention. The supplementation regimen with concurrent heart medication use was considered safe in our research. More studies are warranted to confirm the dose and time-related effectiveness and safety of NK and RYR, either alone or combined, in patients with existing cardiovascular complications, and to explore the mechanism through which the supplementation could exert antithrombotic effects.

## Data availability statement

The original contributions presented in the study are included in the article/Supplementary material, further inquiries can be directed to the corresponding authors.

## Ethics statement

The studies involving humans were approved by the Ethics Committee of Qingdao Center for Disease Control and Prevention. The studies were conducted in accordance with the local legislation and institutional requirements. The participants provided their written informed consent to participate in this study. Written informed consent was obtained from the individual(s) for the publication of any potentially identifiable images or data included in this article.

## Author contributions

ML: Conceptualization, Data curation, Formal analysis, Investigation, Methodology, Resources, Writing – original draft, Writing – review & editing. ZX: Data curation, Formal analysis, Visualization, Writing – original draft, Writing – review & editing. ZW: Investigation, Project administration, Writing – original draft. DW: Data curation, Formal analysis, Writing – review & editing. MY: Formal analysis, Writing – original draft. HLi: Investigation, Project administration, Writing – original draft. WZ: Investigation, Project administration, Writing – original draft. RH: Conceptualization, Writing – review & editing. HC: Investigation, Writing – original draft. PG: Investigation, Writing – original draft. ZL: Conceptualization, Resources, Supervision, Writing – review & editing. HLia: Conceptualization, Funding acquisition, Investigation, Methodology, Project administration, Writing – review & editing.
