# Supplementary material for: Lipid-lowering, antihypertensive, and antithrombotic effects of nattokinase combined with red yeast rice in patients with stable coronary artery disease: a randomized, double-blinded, placebo-controlled trial
Source: Front Nutr. 2024 May 15;11:1380727. doi: 10.3389/fnut.2024.1380727 (PMC11133624; doi:10.3389/fnut.2024.1380727)
Supplement: Supplementary file 1 [file Table_1.docx]

Supplementary Material

# Supplementary Tables

# Supplemental Table 1. Comparison of baseline characteristics between participants who completed the study versus who were lost during the trial.

|  | Completed | Dropped | p value* |
| --- | --- | --- | --- |
| n | 178 | 11 |  |
| ***General*** |  |  |  |
| Men (%) | 71 (39.9%) | 4 (36.4%) | 1 |
| Age (years) | 62.2 (8.84) | 62.9 (6.46) | 0.9613† |
| BMI (kg/m^2^) | 26.1 (2.80) | 25.1 (1.96) | 0.4781† |
| CAD family history (%) | 65 (36.5%) | 8 (72.7%) | **0.0380** |
| Drinkers (%) | 34 (19.1%) | 2 (18.2%) | 1 |
| Smokers (%) | 37 (20.8%) | 3 (27.3%) | 0.8959 |
| ***Biochemical*** |  |  |  |
| SBP (mm Hg) | 140 (16.2) | 125 (17.0) | **0.0028** |
| DBP (mm Hg) | 81.0 (10.2) | 72.6 (13.8) | **0.0433**† |
| TG (mmol/L) | 1.72 (0.508) | 1.65 (0.427) | 0.7120† |
| TC (mmol/L) | 5.31 (0.903) | 5.07 (0.837) | 0.4028 |
| HDL-C (mmol/L) | 1.35 (0.276) | 1.29 (0.172) | 0.6721† |
| LDL-C (mmol/L) | 3.21 (0.814) | 3.04 (0.700) | 0.3371 |
| Glucose | 6.27 (1.70) | 5.90 (2.11) | 0.1539† |
| ***Medications*** |  |  |  |
| Statin (%) | 108 (60.7%) | 1 (9.09%) | **0.0023** |
| Aspirin (%) | 42 (23.6%) | 0 (0%) | 0.1462 |
| β Blocker (%) | 56 (31.5%) | 0 (0%) | 0.0605 |
| ACE Inhibitor (%) | 38 (21.3%) | 0 (0%) | 0.1846 |

* For continuous variables p value among groups analyzed by Student's t-test if normally distributed, or Wilcoxon rank-sum test if non-normal (p value marked †). Chi-square test for categorical variables.

**Supplemental Table 2.** Diet and exercise of participants at baseline and endpoints.

| Diet and Exercise | Treatment | Baseline (T0) | 90 Days (T1) | p value† | Change (T1-T0) | |
| --- | --- | --- | --- | --- | --- | --- |
| Energy (Kcal/d) | NK-RYR | 1670 (412) | 1670 (340) | *0.9993* | -0.0682 (415) |  |
|  | NK | 1710 (407) | 1680 (386) | *0.7238* | -23.6 (299) |  |
|  | RYR | 1670 (390) | 1640 (391) | *0.6514* | -33.0 (330) |  |
|  | Placebo | 1720 (379) | 1800 (323) | *0.2749* | 85.5 (427) |  |
|  | *p value** | *0.9234* | *0.1723* |  | *0.4362* |  |
| Protein  (g/d) | NK-RYR | 67.0 (19.4) | 65.3 (21.6) | *0.7024* | -1.68 (25.0) |  |
|  | NK | 64.0 (19.9) | 68.1 (19.4) | *0.3638* | 4.13 (18.4) |  |
|  | RYR | 63.5 (19.8) | 65.6 (20.5) | *0.6782* | 2.04 (21.2) |  |
|  | Placebo | 67.2 (17.9) | 64.1 (19.0) | *0.444* | -3.14 (21.8) |  |
|  | *p value** | *0.7117* | *0.8343* |  | *0.3917* |  |
| Fat  (g/d) | NK-RYR | 76.7 (21.4) | 76.6 (17.1) | *0.7862* | -0.105 (24.6) |  |
|  | NK | 77.9 (22.6) | 79.9 (21.8) | *0.9323* | 1.99 (23.2) |  |
|  | RYR | 77.4 (19.9) | 77.1 (21.3) | *0.8226* | -0.327 (21.9) |  |
|  | Placebo | 79.2 (18.4) | 81.7 (23.5) | *0.6197* | 2.49 (24.6) |  |
|  | *p value** | *0.9532* | *0.6349* |  | *0.9196* |  |
| Carbohydrates  (g/d) | NK-RYR | 177 (63.9) | 180 (58.3) | *0.8742* | 2.07 (79.0) |  |
|  | NK | 181 (70.9) | 173 (59.3) | *0.6064* | -7.38 (62.2) |  |
|  | RYR | 178 (61.5) | 170 (57.3) | *0.4864* | -8.22 (45.1) |  |
|  | Placebo | 175 (60.6) | 196 (54.6) | *0.1146* | 20.3 (68.0) |  |
|  | *p value** | *0.9858* | *0.1651* |  | *0.1415* |  |
| Exercise Minutes  (min/d) | NK-RYR | 44.3 (25.5) | 42.3 (21.8) | *0.7640* | -2.05 (15.0) |  |
|  | NK | 51.4 (25.9) | 50.7 (24.3) | *0.9578* | -0.714 (12.4) |  |
|  | RYR | 46.5 (23.5) | 46.5 (20.2) | *0.8482* | 0 (12.0) |  |
|  | Placebo | 44.6 (23.4) | 43.9 (20.2) | *0.8258* | -0.732 (10.6) |  |
|  | *p value** | *0.6377* | *0.4121* |  | *0.8799* |  |

* p value among groups analyzed by Kruskal-Wallis considering non-normal distributions in all parameters;

† p value between baseline and 90 days of intervention analyzed by Wilcoxon test, adjusted using Benjamini-Hochberg method

# Supplemental Table 3. Baseline characteristics among participants whose coagulation markers ELISA were measured

|  | **Ctrl (N=23)** | **NK+RR (N=27)** | **NK (N=27)** | **RR (N=31)** | **Total (N=108)** | **P-value*** |
| --- | --- | --- | --- | --- | --- | --- |
| **Age** |  |  |  |  |  |  |
| Mean (SD) | 62.4 (8.51) | 64.7 (8.66) | 60.7 (10.3) | 60.5 (9.19) | 62.0 (9.25) | 0.4152† |
| **Sex** |  |  |  |  |  |  |
| Men (%) | 14 (60.9%) | 10 (37.0%) | 8 (29.6%) | 16 (51.6%) | 48 (44.4%) | 0.1042 |
| Women (%) | 9 (39.1%) | 17 (63.0%) | 19 (70.4%) | 15 (48.4%) | 60 (55.6%) |  |
| **Alcohol Consumption** |  |  |  |  |  |  |
| No | 20 (87.0%) | 19 (70.4%) | 22 (81.5%) | 25 (80.6%) | 86 (79.6%) | 0.5193 |
| Yes | 3 (13.0%) | 8 (29.6%) | 5 (18.5%) | 6 (19.4%) | 22 (20.4%) |  |
| **Smoking** |  |  |  |  |  |  |
| No | 19 (82.6%) | 18 (66.7%) | 21 (77.8%) | 27 (87.1%) | 85 (78.7%) | 0.2770 |
| Yes | 4 (17.4%) | 9 (33.3%) | 6 (22.2%) | 4 (12.9%) | 23 (21.3%) |  |
| **BMI** |  |  |  |  |  |  |
| Mean (SD) | 24.6 (3.12) | 25.4 (2.92) | 25.7 (2.23) | 26.4 (2.67) | 25.6 (2.78) | 0.1138† |
| **SBP(mmHg)** |  |  |  |  |  |  |
| Mean (SD) | 139 (15.8) | 140 (20.4) | 143 (13.0) | 139 (15.8) | 140 (16.3) | 0.7367 |
| **DBP(mmHg)** |  |  |  |  |  |  |
| Mean (SD) | 78.3 (12.6) | 83.5 (12.0) | 83.9 (7.38) | 79.2 (8.41) | 81.3 (10.3) | 0.1030† |
| **TG (mmHg)** |  |  |  |  |  |  |
| Mean (SD) | 1.83 (0.502) | 1.68 (0.447) | 1.76 (0.432) | 1.81 (0.646) | 1.77 (0.516) | 0.7111† |
| **TC (mmHg)** |  |  |  |  |  |  |
| Mean (SD) | 5.33 (0.690) | 5.48 (0.823) | 5.11 (0.934) | 5.12 (1.07) | 5.25 (0.904) | 0.3709 |
| **HDL-C (mmol/L)** |  |  |  |  |  |  |
| Mean (SD) | 1.42 (0.287) | 1.36 (0.184) | 1.30 (0.354) | 1.31 (0.339) | 1.34 (0.300) | 0.1226† |
| **LDL-C(mmol/L)** |  |  |  |  |  |  |
| Mean (SD) | 3.21 (0.443) | 3.14 (0.953) | 3.24 (0.864) | 3.17 (0.794) | 3.19 (0.788) | 0.9669 |
| **Blood Glucose (mmol/L)** |  |  |  |  |  |  |
| Mean (SD) | 6.01 (2.50) | 6.20 (1.56) | 6.00 (1.34) | 6.94 (1.62) | 6.32 (1.79) | 0.0003† |

* For continuous variables p value among groups analyzed by ANOVA if normally distributed, or Kruskal-Wallis if non-normal (p-value marked †). Chi-square test for categorical variables.

**Supplemental Table 4.** Change of outcome indicators after 90-day intervention among participants whose coagulation markers ELISA were measured

| **Change** | **Ctrl (N=23)** | **NK+RR (N=27)** | **NK (N=27)** | **RR (N=31)** | **Total (N=108)** | **P-value*** |
| --- | --- | --- | --- | --- | --- | --- |
| **SBP(mmHg)** | |  |  |  |  |  |
| Mean (SD) | -3.96 (4.17) | -9.00 (8.71) | -7.44 (7.07) | -6.42 (8.61) | -6.80 (7.62) | 0.1246 |
| **DBP(mmHg)** | |  |  |  |  |  |
| Mean (SD) | -1.09 (5.35) | -7.56 (7.89) | -0.148 (7.67) | 1.68 (8.54) | -1.68 (8.27) | < 1e-04† |
| **TG(mmol/L)** |  |  |  |  |  |  |
| Mean (SD) | -0.161 (0.402) | -0.346 (0.462) | -0.193 (0.245) | -0.248 (0.541) | -0.240 (0.432) | 0.2619† |
| **TC(mmol/L)** | |  |  |  |  |  |
| Mean (SD) | -0.024 (0.103) | -0.745 (0.502) | -0.329 (0.692) | -0.407 (0.785) | -0.391 (0.644) | < 1e-04 |
| **HDL-C(mmol/L)** | |  |  |  |  |  |
| Mean (SD) | 0.072 (0.164) | 0.181 (0.232) | 0.043 (0.351) | 0.105 (0.288) | 0.102 (0.273) | 0.2847† |
| **LDL-C(mmol/L)** | |  |  |  |  |  |
| Mean (SD) | -0.009 (0.0986) | -0.584 (0.583) | -0.373 (0.444) | -0.579 (0.641) | -0.407 (0.547) | < 1e-04 |
| **Blood Glucose(mmol/L)** | |  |  |  |  |  |
| Mean (SD) | -0.010 (0.220) | -0.218 (0.816) | -0.878 (1.06) | -1.02 (1.51) | -0.569 (1.13) | < 1e-04† |
| **CK(mmol/L)** | |  |  |  |  |  |
| Mean (SD) | -12.2 (10.0) | -8.93 (13.3) | -14.5 (16.8) | -13.1 (17.5) | -12.2 (14.9) | 0.5646 |
| **LDH(mmol/L)** | |  |  |  |  |  |
| Mean (SD) | -13.5 (19.1) | -34.3 (31.3) | -26.8 (16.9) | -10.1 (29.0) | -21.1 (26.8) | 0.0074 |

* For continuous variables p value among groups analyzed by ANOVA if normally distributed, or Kruskal-Wallis if non-normal (p-value marked †). Chi-square test for categorical variables.

**Supplemental Table 5.** Change of outcome indicators among participants by subgroups of statin use or not.

|  | **No Statin Use** | | | | | | |  | **Concurrently taking Statin** | | | | | | |
| --- | --- | --- | --- | --- | --- | --- | --- | --- | --- | --- | --- | --- | --- | --- | --- |
| **Biomarkers** |  | **Ctrl** | **NK+RR** | **NK** | **RR** | **Total** | **P-value*** |  |  | **Ctrl** | **NK+RR** | **NK** | **RR** | **Total** | **P-value*** |
| **Change** |  | **(N=15)** | **(N=16)** | **(N=19)** | **(N=20)** | **(N=70)** |  |  |  | **(N=26)** | **(N=28)** | **(N=23)** | **(N=31)** | **(N=108)** |  |
| **Heart Rate** | Mean (SD) | -2.73 (6.34) | -7.00 (5.97) | -4.05 (4.60) | -4.25 (4.42) | -4.50 (5.39) | 0.1546 |  | Mean (SD) | -0.923 (4.65) | -4.61 (5.10) * | -2.26 (3.91) | -2.87 (4.42) | -2.72 (4.69) | **0.0289** |
| **SBP** | Mean (SD) | -5.47 (5.73) | -9.44 (7.62) | -3.05 (6.47) | -4.85 (8.88) | -5.54 (7.57) | 0.0882 |  | Mean (SD) | -2.81 (3.01) | -9.54 (7.18) * | -9.04 (6.94) * | -8.29 (8.69) * | -7.45 (7.30) | **0.0018** |
| **DBP** | Mean (SD) | 0.733 (5.85) | -5.88 (9.70)* | 3.53 (6.76) | 2.20 (9.32) | 0.400 (8.72) | **0.0078**† |  | Mean (SD) | -0.731 (5.71) | -8.25 (6.13) * | -1.13 (7.39) | 1.10 (7.43) | -2.24 (7.57) | **< 1e-04**† |
| **TG** | Mean (SD) | -0.0827 (0.478) | -0.277 (0.386) | -0.157 (0.244) | 0.185 (0.425) | -0.0709 (0.418) | **0.0042**† |  | Mean (SD) | -0.129 (0.443) | -0.458 (0.490) * | -0.301 (0.357) | -0.319 (0.616) | -0.305 (0.503) | 0.0860† |
| **TC** | Mean (SD) | -0.0553 (0.0846) | -0.535 (0.533) * | -0.299 (0.749) | -0.382 (0.826) | -0.324 (0.652) | **0.0440** |  | Mean (SD) | -0.0388 (0.101) | -0.734 (0.696) * | -0.340 (0.632) * | -0.424 (0.672) * | -0.393 (0.629) | **< 1e-04** |
| **HDL** | Mean (SD) | 0.0287 (0.115) | 0.0938 (0.227) | -0.00368 (0.162) | 0.0660 (0.282) | 0.0454 (0.210) | 0.5461† |  | Mean (SD) | 0.0804 (0.178) | 0.252 (0.209) * | 0.0165 (0.408) | 0.127 (0.242) | 0.125 (0.276) | **0.0086**† |
| **LDL** | Mean (SD) | 0.0127 (0.0892) | -0.497 (0.677) * | -0.309 (0.479)* | -0.474 (0.613) * | -0.330 (0.550) | **0.0053** |  | Mean (SD) | -0.0227 (0.0903) | -0.560 (0.563) * | -0.387 (0.532) * | -0.580 (0.640) * | -0.400 (0.553) | **< 1e-04** |
| **Glucose** | Mean (SD) | 0.0127 (0.0653) | -0.226 (0.682) | -0.504 (0.536)* | -0.913 (1.49) * | -0.447 (0.952) | **0.0014**† |  | Mean (SD) | -0.00385 (0.211) | -0.348 (0.830) * | -1.03 (1.30) * | -1.23 (1.37) * | -0.663 (1.14) | **< 1e-04**† |
| **CK** | Mean (SD) | -8.73 (15.2) | -4.94 (16.1) | -11.3 (14.5) | -15.1 (18.7) | -10.4 (16.4) | 0.3079 |  | Mean (SD) | -12.6 (13.5) | -11.5 (9.99) | -15.8 (19.4) | -12.9 (18.6) | -13.1 (15.7) | 0.8672 |
| **LDH** | Mean (SD) | -12.7 (24.3) | -37.0 (31.6) * | -26.7 (17.1) | -8.10 (30.0) | -20.7 (28.1) | **0.0116** |  | Mean (SD) | -9.81 (19.8) | -24.6 (34.6) | -26.1 (16.5) * | -15.9 (28.4) | -18.9 (26.8) | 0.0949 |
| **UA** | Mean (SD) | 0.733 (8.34) | -2.31 (40.0) | -10.2 (16.2) * | -21.0 (25.7) * | -9.13 (26.2) | 0.0578 |  | Mean (SD) | 2.00 (7.95) | -13.2 (63.7) | -4.30 (20.2) * | -26.6 (33.3) * | -11.5 (39.4) | **0.0003** |
| **CRP** | Mean (SD) | -0.131 (0.368) | -0.470 (0.667) | -0.319 (0.355) | 0.340 (0.871) | -0.125 (0.683) | **0.0035** |  | Mean (SD) | -0.126 (0.326) | -0.576 (0.904) * | -0.428 (0.462) * | -0.0645 (1.00) | -0.289 (0.777) | **0.0075** |

* p value among groups analyzed by ANOVA if normally distributed, or Kruskal-Wallis if non-normal (p-value marked †).
